# An Insight into the Impact of Thermal Process on Dissolution Profile and Physical Characteristics of Theophylline Tablets Made through 3D Printing Compared to Conventional Methods

**DOI:** 10.3390/biomedicines10061335

**Published:** 2022-06-06

**Authors:** Nour Nashed, Matthew Lam, Taravat Ghafourian, Lluis Pausas, Memory Jiri, Mridul Majumder, Ali Nokhodchi

**Affiliations:** 1 Pharmaceutics Research Laboratory, Arundel Building, School of Life Sciences, University of Sussex, Brighton BN1 9QJ, UK; n.nashed@sussex.ac.uk (N.N.); m.lam@sussex.ac.uk (M.L.); 2 School of Life Sciences, Faculty of Creative Arts, Technologies and Science, University of Bedfordshire, Luton LU1 3JU, UK; tara.ghafourian@beds.ac.uk; 3 M2M Pharmaceuticals Ltd., The Gateway Building, 1 Collegiate Square, Thames Valley Science Park, Reading RG2 9LH, UK; lluis.pausas@m2mpharma.com (L.P.); memory.jiri@m2mpharma.com (M.J.); mridul.majumder@m2mpharma.com (M.M.)

**Keywords:** 3D printing, hot-melt extrusion, manufacturing method, fused deposition modeling, drug release, porosity, density

## Abstract

The dissolution profile is of great importance in drug delivery and is affected by the manufacturing method. Thus, it is important to study the influence of the thermal process on drug release in emerging technologies such as 3D printing-fused deposition modeling (FDM). For this purpose, the characteristics of 3D printed tablets were compared to those of tablets prepared by other thermal methods such as hot-melt extrusion (HME) and non-thermal methods such as physical mixture (PM). Theophylline was used as a drug model and blends of ethyl cellulose (EC) and hydroxypropyl cellulose (HPC) were used as a matrix former. The solid state of the drug in all formulations was investigated by differential scanning calorimetry, X-ray powder diffraction, and Fourier-transformed infrared spectroscopy. All studied tablets had the same weight and surface area/volume (SA/V). Dissolution data showed that, for some formulations, printed tablets interestingly had a faster release profile despite having the highest hardness values (>550 N) compared to HME and PM tablets. Porosity investigations showed that 100% infill printed tablets had the highest porosity (~20%) compared to HME (<10%) and PM tablets (≤11%). True density records were the lowest in printed tablets (~1.22 g/m^3^) compared to tablets made from both HME and PM methods (~1.26 g/m^3^), reflecting the possible increase in polymer specific volume while printing. This increase in the volume of polymer network may accelerate water and drug diffusion from/within the matrix. Thus, it is a misconception that the 3D printing process will always retard drug release based on increased tablet hardness. Hardness, porosity, density, solid-state of the drug, SA/V, weight, and formulation components are all factors contributing to the release profile where the total balance can either slow down or accelerate the release profile.

## 1. Introduction

Fused deposition modeling (FDM) is one of the 3D printing techniques used to fabricate solid dosage forms. FDM has gained great interest as it can contribute to the potential future of personalized medicine and digital pharmacy [1]. The filaments for FDM can be prepared by hot-melt extrusion (HME) with a suitable diameter to ensure compatibility with the printing nozzle. High temperatures are applied in both technologies (FDM and HME), though it is higher in FDM than in HME, causing great concern for the stability of dosage forms. Thus, the techniques are only suitable for thermostable excipients and active pharmaceutical ingredients (APIs) [1]. Solid dispersion formulations are the most common type of formulations made by HME and FDM where thermoplastic polymers are usually used for this purpose. Thus, determination of the melting point, glass transition, and thermal stability are some of the important pre-formulation tests. In addition, mechanical characteristics should be investigated before selecting formulations to be printed [1]. Filaments to be used in FDM need to have adequate elasticity to withstand coiling around spools and extrusion while printing without rupture or deformation. Plasticizers are usually used to improve the mechanical properties of the filaments. Moreover, certain polymer blends can induce an internal plasticization effect, which improves the mechanical properties and printability of filaments in FDM [2].

Polymer blends have been widely used for oral drug delivery to modulate drug release since it can be difficult to reach the proposed release rate using a single polymer. Another advantage of the polymer blend is that it can have better printability compared to a single polymer filament in FDM. Polymers that are blended need to be compatible and miscible, especially in amorphous solid dispersion formulations, to avoid phase separation, which can affect the drug dissolution profile. Hydroxypropyl cellulose (HPC) and ethyl cellulose (EC) were found to be compatible blends [3] and have been used in several studies [4,5]. Both polymers are thermostable and compatible with HME and FDM [1].

This study focuses on the effect of the FDM on drug release compared to other manufacturing methods. It has been believed that FDM can reduce drug release because of the drug being entrapped among the polymer network, and the increased hardness of tablets [6,7]. When it comes to temperature, the focus is usually on stability and chemical degradation. However, several studies mentioned that polymers are susceptible to changes in their specific volume, free volume, and density after heating/cooling, which can alter water permeability through the polymeric matrix and thus the dissolution rate [8,9,10,11]. Therefore, to this end, it is important to investigate the physical properties of FDM 3D printed tablets and how FDM thermal processing can modulate drug release. In FDM, tablets are built layer over layer with no compression forces as in conventional methods, which may affect the porosity of the printed tablets. In addition, materials are exposed to a dual heating process, one is while making filaments (HME) and the another is during printing. Thus, a comparative study of the single-thermal method (HME) and non-thermal method (PM) would help acquire a wider understanding of the physical changes in FDM.

To rule out any thermal or chemical instability, theophylline (model drug), HPC and EC were selected for this study as they are thermostable and compatible [4,5]. Since the expected changes in free volume under high temperatures can change permeability and diffusion characteristics of the polymer, swelling behavior might be affected [11,12]. Thus, two grades of HPC (JF for extended release and EF for immediate release) were selected for this study for a clearer understanding of any changes in swelling behavior due to elevated temperatures. To this end, six formulations using blends of HPC, EC, and theophylline were studied. Tablets of each formulation were prepared by three different techniques (physical mixture, HME, and FDM 3D printing). For an accurate comparison, all tablets were designed to have the same surface area/volume (SA/V) and weight. Hardness, true density, and porosity were recorded to aid the investigation of the effect of the manufacturing method on drug release.

## 2. Materials and Methods

### 2.1. Materials

Theophylline anhydrous with a purity of >99% was purchased from Fisher Scientific (Loughborough, UK) and was used as a model drug with a high melting point of around 273 °C. Ethylcellulose (EC, Ethocel 10 FP) was obtained from Colorcon Ltd. (Dartford, UK). Two grades of hydroxypropyl cellulose (HPC, klucelTM EF and klucelTM JF) were provided by Ashland Inc. (Rotterdam, The Netherlands). All materials were used as received.

### 2.2. Selection of Formulations to Be Prepared by the Three Methods (PM, HME, and FDM)

In order to rule out issues concerning chemical instability, the current study used materials such as theophylline, EC, and two grades of HPC (EF and JF) as they are thermoplastic and can withstand very high temperatures without degrading [4,5]. Four ratios of EC and HPC blends were used as the matrix former, where the composition percentage is shown in Table 1. The percentage of theophylline was constant in all formulations (30% *w/w*). EC was too brittle to be printed, therefore, in formulations with a high percentage of EC (F1 and F5), dibutyl sebacate (DBS) with a concentration of 5% *w/w* was used to plasticize EC. DBS was mixed manually with EC powders and left overnight for better sorption of liquid plasticizer in EC chains. Then, the next day, the plasticized EC was ready for use. After preliminary investigations, only printable formulations were selected for further studies, where tablets of successful formulations (F1–F3 and F6–F8) were prepared by PM, HME, and FDM methods.

### 2.3. Preparation of Tablets from Physical Mixture (PM Tablets)

Tablets of successful formulations mentioned in Table 1 were prepared by manually mixing powders using a pestle and mortar to break down any agglomerates. A specific amount of the physical mixture (333.33 mg) equivalent to 100 mg of theophylline was compressed by a manual tablet press (Model MTCM-ɪ, Globe Pharma, New Brunswick, NJ, USA) equipped with 10 mm diameter concave punches. Compression was done at 150 bars with 10 s dwell time.

### 2.4. Preparation of Tablets from HME (HME Tablets)

The admixture of excipients and API was mixed manually in the same manner as PM tablets but with an additional process where it was fed manually to a 10-mm twin-screw extruder L/D 20 (assembled by Point1 Controls/R Controls, Stoke-on-Trent, UK) at a screw speed of 50 rpm. The temperature of the feed zone was set at 110 °C to prevent drug accumulation, and the temperature for the rest of the zones (including die) was set at 150 °C. This temperature was selected to exceed the Tg of formulations without reaching the melting point of the materials used in the formulations. This is to allow the collection of filaments with intact shapes to be achieved. Filaments were collected using a winder. After cooling, filaments were cut manually using a pair of scissors into small pieces around 2 mm. Then, an amount of 5 g was transferred into a ball mill (PM 100, Retsch GmbH, Haan, Germany) and ground for 4 min at 400 rpm to convert 2 mm filaments into powder form for tableting. Powders were collected and kept in tightly closed containers. The obtained powders were compressed into tablets in the same way as PM tablets. Compression was carried out at 150 bars with 10 s dwell time. The nominal weight of each tablet was 333.33 mg equivalent to 100 mg of theophylline.

### 2.5. Preparation of Tablets Printed by FDM (Printed Tablets)

Filaments prepared by HME (Section 2.4) were used as feedstock for printing. The diameter of collected HME filaments was between 1.65 and 1.8 mm to fit the diameter of the printing nozzle (1.75 mm). The computer-aided design (CAD) model of a cylindrical tablet was designed online using TinkerCAD and then exported as an .STL file which was then imported to the software of Makerbot Replicator 2X printer (Makerbot Inc., New York, NY, USA). Printed tablets had a height of 5.45 mm and a diameter of 8.8 mm. Based on the preliminary experiments, those dimensions were selected for two reasons; the first is to obtain the same weight of tablets prepared by other methods (333.33 mg) and the second is to obtain very close SA/V to the other tablets produced by HME and PM methods. This can make the comparison more accurate as weight and SA/V can have an impact on drug release. Printing settings were as follows: layer height (0.2 mm), infill density of 100%, the number of shells (2), printing speed of 90 mm/s, bed temperature (50 °C), and nozzle temperature (220 °C).

### 2.6. Differential Scanning Calorimetry Analysis (DSC)

DSC helps determine the solid-state of used materials and their thermal transitions, glass transition, and melting point, which helps in setting HME and printing conditions. DSC can also detect any shifts in glass transition or melting points due to intermolecular interactions among components of the formulation. Thus, DSC was used to investigate the thermal stability and miscibility/compatibility of the materials used in making tablets in HME and 3D printing. In this experiment, glass transition and the melting point of EC, HPC, theophylline, PM tablets, HME tablets, and printed tablets were determined by a DSC 4000 system (Perkin Elmer, Waltham, MA, USA). Around 10 mg of each material or formulation was placed into crucible aluminum pans, and samples were scanned from 25 °C to 350 °C at a scanning rate of 10 °C/min under nitrogen gas as the purged gas with a flow rate of 20 mL/min. The obtained DSC traces were analyzed using Pyris software (Perkin Elmer, Waltham, MA, USA).

### 2.7. Fourier-Transform Infrared Spectroscopy (FTIR)

To investigate any changes or interactions at the molecular level, the samples were subjected to FTIR analysis (Spectrum One, PerkinElmer, Waltham, MA, USA). The scanning range was set from 4000 cm^−1^ to 650 cm^−1^. The powdered samples (a few mg) were placed on the ATR crystal slit and were pressed up to around 110 N. The resultant spectrum was an average of 4 scans

### 2.8. X-ray Powder Diffraction Analysis (XRPD)

To investigate the effect of extrusion and 3D printing on the crystallinity of the formulations, diffractograms of theophylline, EC, HPC, physical mixture tablets, HME tablets, and printed tablets were determined by a Siemens D500 X-ray diffractometer (Siemens, Munich, Germany) using copper Rf radiation at 40 KV voltage and 30 mA. Data were recorded between 2θ of 5°to 500 at a step width of 0.01° and 1 s time count. Using MATLAB R2021a, crystallinity was calculated from the obtained diffractograms based on the following equation:

Crystallinity = (area of crystalline Peak)/(Area of all peaks (crystalline and amorphous)) × 100

### 2.9. Characteristics of Prepared Tablets

For better comparison and accurate observations, several characteristics related to tablets, such as hardness, porosity, and SA/V ratio, were considered. The hardness of tablets was estimated using texture analysis (Stable Micro Systems, Godalming, UK). Surface area and volume were calculated based on the dimensions of tablets measured by a Vernier caliper. For HME and PM tablets, the concave shape was considered in the calculations. To calculate the approximate porosity of the obtained tablets, the apparent density of the tablets and the true density were determined. The average apparent density for 3 tablets of each formulation prepared by each method was calculated based on their values of volume and weight. Since the thermal process can alter the specific volume of the polymer, and thus its density [13], the true density of each type of tablet was calculated using a helium pycnometer. The density of physical mixture powders, ground filaments, and ground printlets were considered the true density for PM, HME, and printed tablets, respectively. Then, the porosity of all tablets was calculated by employing the following equation:

Porosity% = 1 − (apparent density)/(true density) × 100

### 2.10. In Vitro Dissolution and Release Kinetic Model Studies

Dissolution tests were carried out for tablets (*n* = 3) under sink conditions using a USP type II paddle apparatus (708-DS Dissolution Apparatus, Agilent Technologies, Santa Clara, CA, USA) attached to a UV spectrophotometer (Cary 60 UV-Vis, Agilent Technologies). Vessels were filled with 900 mL of deionized water. The temperature was set at 37 ± 1 °C, and the paddle rotation speed was set at 50 rpm. The duration of the test was 16 h, with readings taken at different time intervals. Absorbance was taken every 10 min for the first two hours, then 30 min for the next two hours, and an hour interval until 16 h. The wavelength used to measure the absorbance of theophylline was 271 nm [14]. Drug release profiles were plotted as a percentage of cumulative drug release versus time (h).

Dissolution efficiency (DE%) and release kinetics for all tablets prepared by the three methods were calculated using DDSolver software (an add-in program in Microsoft Excel) [15]. Data were fitted to several drug release kinetic models, namely zero-order, first-order, Higuchi, and Korsmeyer–Peppas. R2adj was used to get the best-fitted model, where the higher R2adj value is, it is an indication of the best fitting model. For Korsmeyer–Peppas, the *n* value can indicate the mechanism of drug release if the drug release is considered up to 60% [6,16]. When *n* approaches 0.5, drug release is governed by diffusion (Fickian model). If *n* approaches 1, swelling is the main release mechanism, and the drug release follows zero-order kinetics (non-Fickian model). For 0.5 < *n* < 1, drug release is governed by both diffusion and swelling (anomalous transport) [6,16].

### 2.11. Tablets Water Uptake

Since HPC is a swellable polymer, swelling capacity was investigated to observe if there were any changes in a swelling capacity that could be caused by the heating and cooling processes. Tablets were collected after the end of the dissolution test and the excessive water was removed using paper. Then, tablets were weighed and kept for drying in an oven at 50 ℃ for 24 h. The weight of dried tablets was also recorded. Since part of tablets can dissolve during the dissolution test, water uptake% was calculated using the following equation:

Water uptake% = (W1 − W2)/W2 × 100

where W1 is the weight of tablets at the end of the dissolution test and W2 is the weight of dried tablets [17]. Calculations were done in triplicate for more accuracy.

## 3. Results and Discussion

### 3.1. DSC Studies

**Figure 1:**
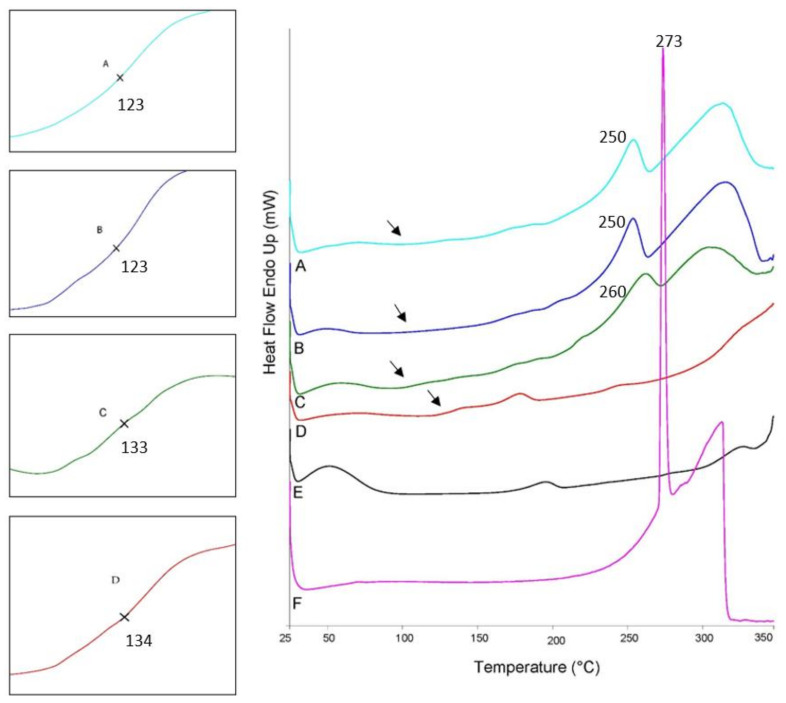
DSC thermograms for raw materials and tablets of F2: (**A**) printed tablets, (**B**) HME tablets, (**C**) PM tablets, (**D**) EC, (**E**) HPC, (**F**) theophylline. Arrows refer to the positions of glass transitions that are shown on a larger scale on the left side of the graph.

Differential scanning calorimetry was carried out in the range from 20 to 350 °C, covering the temperature range used in extrusion (150 °C) and printing (220 °C). Thermograms of HPC and EC (Figure 1) showed very high amorphousness with very low crystallinity reflected by small and wide endothermic peaks at around 195 °C and 178 °C, respectively, which is in accordance with other studies [18,19,20]. Although the glass transition of HPC is mentioned to be between 0–120 °C, according to the manufacturer [21], sometimes it was undetectable in DSC as reported by other studies [4], which was also the case in the current study. DSC traces of EC showed T_g_ at around 134 °C. Endothermic peaks in the range of over 250 °C are likely to account for the thermal degradation of polymers. There was also a wide endothermic peak from 40 to 100 °C, corresponding to the moisture evaporation from both polymers, but it was more obvious in HPC, which is more hygroscopic than EC [19]. Figure 1 also showed that theophylline had a crystalline structure where its thermogram showed a very sharp endothermic peak at around 273 °C, accounting for its melting transition [22]. The degradation of theophylline started immediately after its fusion, which was obvious from the endothermic peaks after the melting transition [22].

Thermograms of the six formulations were very similar as the used polymers and API were the same. Thus, to avoid repetition, the thermogram of just one formulation (F2) in the three manufacturing methods is shown in Figure 1. Thermograms of all formulations showed shifts in melting and glass transitions, indicating interactions among formulation components (Figure 1). Melting of theophylline dropped from 273 °C to around 260 °C in PM tablets, but the reduction in melting peak was more noticeable in the case of formulations obtained via HME or 3D printing (250 °C). The significant reduction in the melting peak of theophylline in HME or printed formulations could be due to better mixing and higher interactions of theophylline with the polymers, which was proved in FTIR results. It should be also mentioned that the melting transitions of polymers overlapped in mixtures and are shown as a very broad endothermic peak from 165 to 191 °C in all formulations.

The glass transition was detected in DSC traces for all formulations (except F8). As the T_g_ of HPC was hardly detectable in DSC, it is hard to tell whether this T_g_ was for EC or the polymer blends. In PM tablets, T_g_ was observed at around 133 °C, while in HME and printed tablets, it shifted to around 123 °C. Although it was hard to detect to which polymer this T_g_ belonged, this shift can confirm the compatibility of polymer blends as found in other studies [3]. In formulation F1, which contained DBS as a plasticizer, the glass transition was detected at around 120 °C in PM tablets and dropped to 110 °C in HME and printed tablets.

### 3.2. FTIR Spectrum

**Figure 2:**
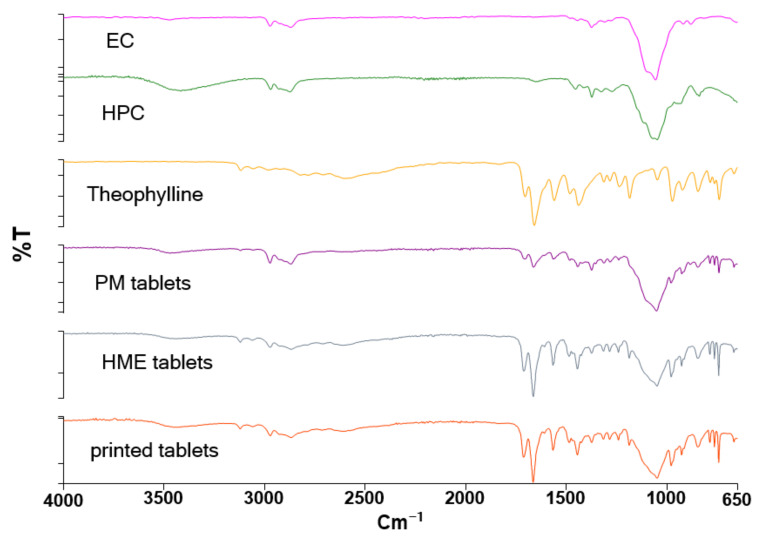
FTIR spectrum of F2 formulations obtained via different methods and raw materials.

FTIR spectrum for all materials used in the preparation of formulations is shown in Figure 2. Concerning theophylline, bands between 1600 cm^−1^ and 1800 cm^−1^ accounted for the stretching of the C=O, C=N, C=C. Bands in the range of 2500 to 3300 cm^−1^ were allotted for stretching N-H and C-H in the imidazole ring. Peaks at 1500 to 1000 cm^−1^ were associated with the stretching and bending C-C, C-N, while the range from 1000 cm^−1^ to 650 cm^−1^ was associated with the low-frequency vibrations (bending) of almost all bonds [23,24,25].

As EC and HPC have a similar molecular structure, both showed a similar FTIR spectrum. The peak in the range of 3000 to 3500 cm^−1^ was an indication of O-H stretching in the pyranose ring [25,26]. Compared to EC, the intensity of the O-H band in HPC was higher, which is probably due to the additional hydroxyl group in the side chain, which is absent in ethyl cellulose. Bands in the range of 3000 to 2500 cm^−1^ accounted for C-H stretching vibration, while the very strong absorption between 1200 and 1000 cm^−1^ mostly belonged to the stretching vibration of C-O bonds in both polymers [25,26].

Although the FTIR spectrum of formulations showed the characteristic peaks of EC, HPC, and theophylline, the spectrum of HME and printed tablets showed higher intensity in the bands of groups involved in hydrogen bonding, O-H (3000–3500 cm^−1^), and C=O (1600–1800 cm^−1^) compared to the spectrum of physical mixture. Due to the efficient mixing in HME, there were more hydrogen bonds among the components in the formulation (EC, HPC, theophylline), which can increase the intensity of FTIR bands of proton donor and acceptor [27]. Thus, the relative intensity of bands at 1600–1800 cm^−1^ to the strongest band at (1000–1200 cm^−1^) was found to be higher in HME and printed tablets compared to PM where it was lower due to the absence of H-bonds. As all formulations had the same components with just different fractions, their FTIR spectrums were similar and the spectrum of just one formulation (F2) is mentioned in Figure 2.

### 3.3. X-ray Powder Diffraction Analysis (XRPD)

**Figure 3:**
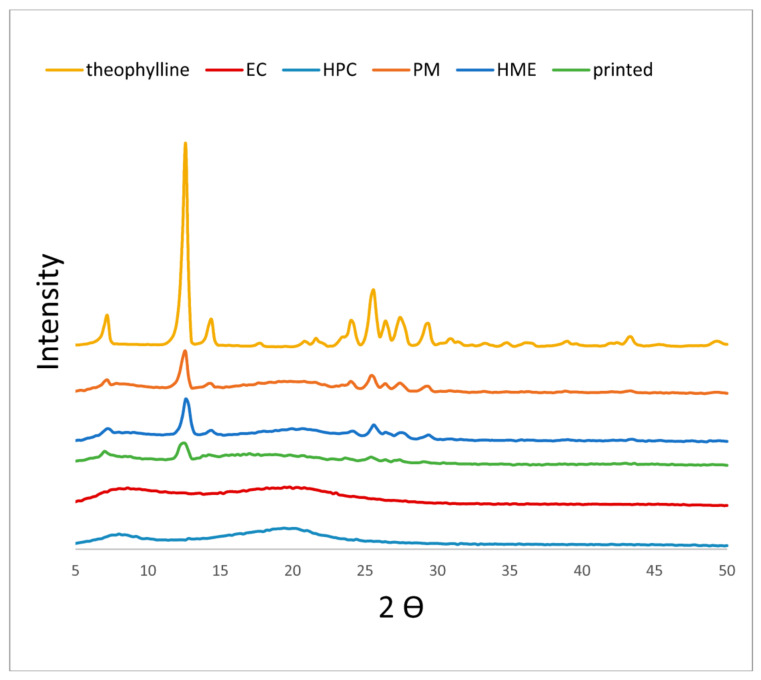
XRPD diffractograms of F2 tablets and raw materials.

X-ray diffractograms of raw materials and PM, HME, and printed tablets were recorded between 2θ of 5 and 50. Only F2 diffractograms are shown in this paper to avoid repetition as all formulations showed similar diffractograms (Figure 3). The diffractogram of pure theophylline showed many characteristic sharp peaks, and the highest one was at around 2θ of 12.6. The diffractograms of EC and HPC had a halo shape with no sharp peaks, indicating an amorphous structure. Unlike other studies [14,28], the diffractograms of all formulations, including 3D printed and HME formulations, demonstrated the existence of theophylline in the crystalline state. This is probably a result of high drug loading (30%) similar to other studies [29,30]. Crystallinity was calculated to investigate any changes in crystallinity percentage due to the manufacturing method. Table 2 shows crystallinity percentage values for a single run for each formulation prepared with different methods. All formulations showed lower crystallinity than theophylline because of being mixed with amorphous polymers. This decrease in crystallinity agreed with its percentage in all formulations (30%). However, the changes in crystallinity % among the PM, HME, and printed tablets were insignificant. Based on these data, it could be concluded that PM, HME, and printed tablets have similar crystallinity. The decrease in peak intensity in printed samples has nothing to tell because of the variations in XRPD samples’ weight and particle size. Thus, the area under the curve was used instead for accurate analyses of XRPD data.

### 3.4. Characteristics of Prepared Tablets by Various Methods

Preliminary investigation showed that six out of the eight proposed formulations were printable and selected for the comparison study. Because changes in weight and SA/V can alter dissolution profile, all produced tablets (PM, HME, and printed tablets) had the same weight and SA/V for accurate comparison and understanding of dissolution behavior (Table 3 and Table 4). Similar to other studies [7], printed tablets had the highest hardness followed by HME tablets and PM tablets (Table 5). This increase in hardness can come from melting and solidification which occurs once in HME and a second time in FDM (as the filament was prepared via HME) resulting in stronger intermolecular bonds in FDM than in HME and PM, respectively.

Table 6 and Table 7 show values of true density and apparent density for all tablets. Despite the minor difference (f_1_ ~ 5%), the true density of printed tablets was less compared to HME and PM ones. This could be justified by the expansion of molten materials under high temperatures (220 °C) followed by a quick temperature drop to room temperature (within less than 3 min), leaving not enough time to restore the polymers’ initial volume before heating [9,13]. In other words, a specific volume of polymers after heating/cooling treatment was higher than their initial volume before heating, associated with a decrease in their true density [9,13,31,32]. This increase in volume is associated with an expansion in the polymer network which may foster water permeability within the matrix and dissolution rate [10,11].

**Figure 4:**
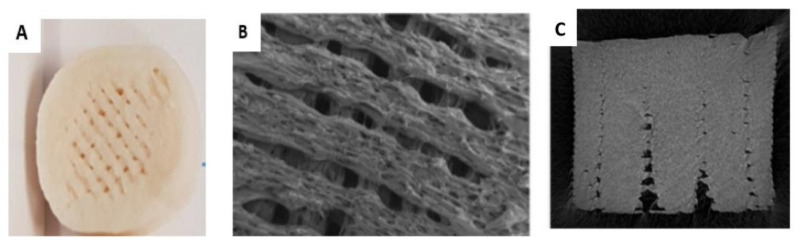
A collection of captures of 100% infill printed tablets showing the gaps within their layers: (**A**) phone picture of the surface (lab work), (**B**) SEM image for the top surface [6], (**C**) X-ray micro-CT image of cross-section [33].

Similar to the data from true density, apparent density values for printed tablets were also the lowest compared to HME and PM, with an average difference factor, f_1_ ~ 19%. The PM and HME had very close apparent density (f_1_ ~ 5%). This can be attributed to the nature of the printing method where tablets are built up layer by layer with no compression forces. Each layer is formed from several threads set beside each other through the synchronization between nozzle onward/backward movements and hot molten discharge from it. This can result in gaps within the layer even with 100% infill (Figure 4). Furthermore, the molten discharge is susceptible to shrinkage while cooling, resulting in larger holes inside the tablet causing a decrease in tablets’ apparent density and increase in their porosity. It should be mentioned that printed tablets, unlike PM and HME tablets, showed floating behaviour for the first 30 min in dissolution, which agrees with apparent density values ≤ 1g/m^3^.

Based on the density values, it could be concluded that printed tablets (100% infill) had the highest porosity (~20%), as shown in Table 8, which agrees with the findings in a recent study by Fanous, Bitar et al. [33]. This increase in porosity was associated with an increase in hardness. In conventional methods, hardness and porosity are correlated to the compression force, where increasing the force can decrease porosity and increase hardness [34]. Thus, hardness and porosity are inversely correlated. However, in the printing method, where tablets are made of layers without any force, hardness seems to be less affected by porosity.

### 3.5. Dissolution Studies

**Figure 5:**
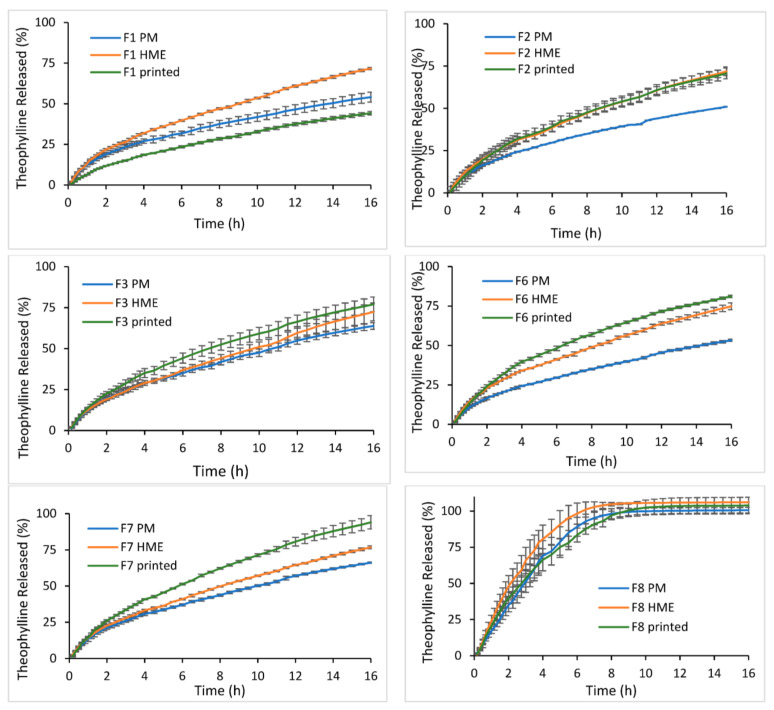
Effect of preparation method on the dissolution profile of theophylline tablets made via physical mixture, HME, and 3D printing.

Dissolution tests were performed for the six formulations prepared by different methods, but the results showed no specific patterns (Figure 5). Physical factors (Section 3.4), hardness, porosity, true density, weight, SA/V, solid-state of theophylline, and composition of formulations were taken into consideration when interpreting the dissolution data.

Generally, an increase in hardness and decrease in porosity tended to result in slower drug release [1,34,35]. For a better understanding of the relationship between the physical properties of the resultant tablets with the dissolution profiles (Figure 5), dissolution efficiency (DE%) was calculated, and the results are shown in Table 9. It is obvious from the DE% and physical properties of the tablets (Table 3, Table 4, Table 5 and Table 8) that no specific correlation could be established. For instance, the hardness of HME tablets (486N–550N) was higher than PM tablets (235 N–326 N), while porosity for both was very close (~10%). So, it was expected that DE% values would follow this order PM > HME tablets. However, this was not the case here as HME tablets showed higher DE% for all formulations compared to PM ones. Printed tablets had the highest porosity (~20%) and highest hardness (>550 N), thus, it was hard to predict the dissolution profile. DE% for printed tablets was unpredictable, where it was the highest in some formulations such as F3, F6, and F7 and was the lowest in F1. Although HME and FDM are thermal processes that may reduce the crystallinity and increase release rate, it was not the case in the current study based on XRPD and DSC results. These findings indicate that other important changes may have taken place in HME and FDM and can influence the dissolution behaviour of the tablets.

**Figure 6:**
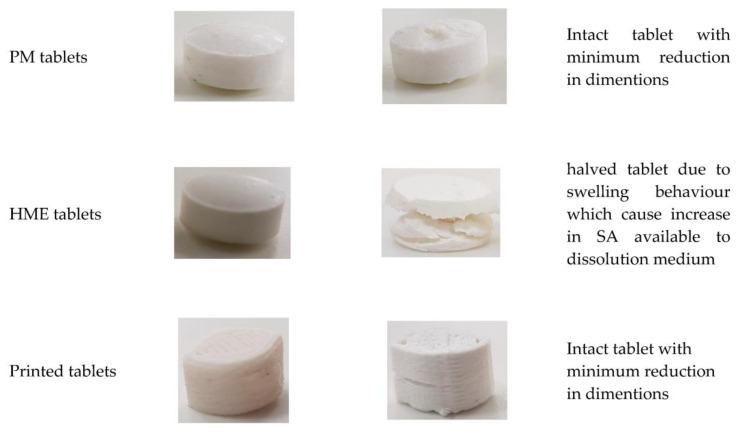
Visual changes to the shape of tablets (F2) after and before dissolution test.

In HME, components were mixed, while melting under high pressure providing dispersive and distributive mixing; thus, the number and type of available and unavailable groups for interaction with the dissolution medium may change [36]. Therefore, two suggestions could justify the high DE% for HME tablets despite their elevated hardness: (1) drug particles are dispersed in the molecular level which can improve their dissolution, and (2) the hydrophilic/hydrophobic balance between polymer blends may change, thus, the dissolution behaviour of extruded formulations [36]. The second suggestion was supported by water uptake data shown in Table 10. There was a noticeable increase in swelling capacity in HME tablets compared to the PM ones. This reflects the higher influence of HPC (hydrophilic) on drug release in HME compared to PM tablets where EC (hydrophobic) was a more dominating release rate. This increase in swelling capacity (swelling pressure) seemed to cause HME tablets to rupture during dissolution and created more SA available for dissolution medium (Figure 6), which is the third suggestion behind the high DE% of HME tablets.

On the contrary, swelling capacity seemed to be reduced in printed tablets of formulations with a high swellable grade of HPC JF (F1, F2, and F3). This change in swelling capacity can be attributed to a reduction in true density. It was discussed in Section 3.4 that the polymer network would expand after heating and fast cooling, leading to higher water permeability, which in turn can alter the swelling behavior of the formulation. Figure 6 shows that printed tablets remained intact during dissolution without any rupturing, as in HME ones.

Printed tablets were made of HME filaments, so their dissolution behavior was expected to be also faster than PM tablets, which was not the case for all formulations. Printed tablets had the highest porosity, lowest true density, and the highest hardness, which have contradicted the impact on dissolution behavior (see Section 3.4). Thus, dissolution behavior followed the balance among those factors, which seemed to be different in each formulation. In F8, DE% of printed was nearly the same as PM, while in F2 it was closer to HME. However, in F3, F6, and F7, DE% of printed tablets was the highest compared to HME and PM tablets. F1 had a completely different pattern, where the effect of hardness and reduced swelling pressure preserved the size and enabled more control on drug release from the printed tablets.

There are other comparative studies by Shi et al. and Zhang et al., whose findings were completely different from the current study [6,7]. They showed that 3D printed tablets had a slow drug release rate, which was attributed to the higher hardness values due to the influence of the FDM process. The authors also mentioned that the inclusion of EC in a high percentage (≥50%) should also result in retarding drug release in HME and printed tablets more than PM tablets. This is due to intermolecular interaction formed with EC during the melting process in the extrusion process, restricting drug release [6,7]. Porosity was not measured in either studies and formulations were different along with greater variation in the hardness, and SA/V between the PM, HME, and printed tablets. Those big variations seem to overcome the effect of heating and fast cooling on the drug release. Thus, the findings were different and difficult to compare with the current study. The conditions of our study were set to have tablets with little variation in weight, SA/V, and hardness and porosity were calculated to get a better understanding of how FDM (as a thermal manufacturing method) can affect drug release rate.

**Figure 7:**
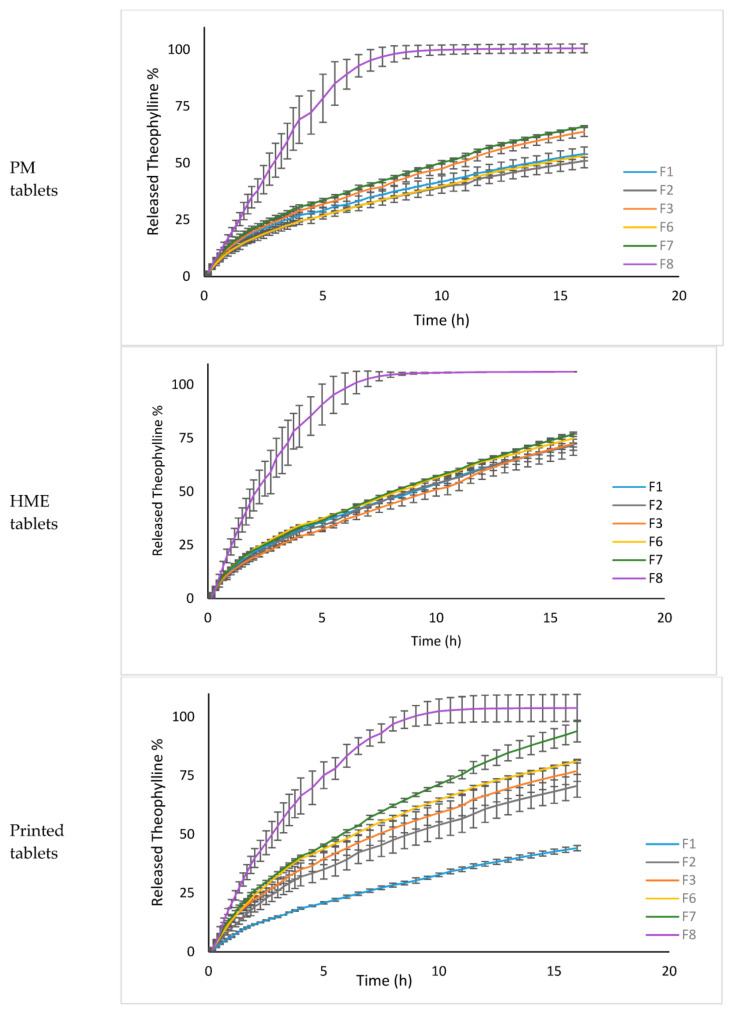
Comparison of the release percentage among the studied formulations in the three preparation methods.

Due to physical changes in the behaviour of polymer mixture (true density, swelling capacity, hardness, and porosity) among different preparation methods, the effect of formulation composition on dissolution profile was also different among the three methods, Figure 7. F8 had nearly the same DE% for printed, HME and PM tablets, 86.87%, 87.98% and 80.25%, respectively. While for other formulations (F1–F7) there was a big difference, with DE% ranges from 26.98 to 58.49%, 42.36 to 47.36% and 32.62 to 42.05% for 3D printed, HME, and PM tablets, respectively. 3D printing tablets had higher sensitivity to any change in formulation. The type of polymer, its amount, and its grade affect drug release. However, in PM tablets, the grade of HPC was found to have nearly no effect on the release of theophylline. Thus, formulations such as F2 and F6 had quite similar DE values with a difference factor (*f*_1_ = 2.94%) and a similarity factor (*f*_2_ = 92.91%). While the influence of HPC grade was sounder in HME method compared to PM method. For instance, the difference between DE% of F3 and F7 was bigger (*f*_1_ = 9.84%) compared to that between F2 and F3 (*f*_1_ = 0.37%).

Overall, this study, for the first time, discussed deeply how high temperatures in 3D printing FDM affects density and release profile. The current study showed the increased porosity of printed tablets due to the printing method which naturally leaves gaps within layers. On top of that, it drew attention to the potential increase in the specific volume of the polymer, which was associated with a decrease in true density. The result of this study compared to the result of other studies showed that FDM 3D printing resulted in changes that were more likely to enhance drug release, especially with hydrophilic formulations. However, if the formulation was more hydrophobic, FDM could help more in slowing down the release rate due to intermolecular interactions with hydrophobic polymers such as EC. This study gives a wider understanding of FDM technology, which can be employed in the best selection of materials based on the proposed drug release rate of designed printed tablets.

### 3.6. Release Kinetics

The release data of theophylline tablets from the six formulations made via PM, HME, and 3D printing tablets were fitted into four kinetics models, zero-order, first-order, Higuchi, and Korsmeyer–Peppas, Table 11. Having prepared tablets by different methods, the drug release seemed to be controlled by diffusion and swelling of the matrix. All prepared tablets fit with the Korsmeyer–Peppas model (R^2^ ≥ 0.99) with *n* values (0.5 < *n* < 1), proving the non-Fickian or anomalous transport. Despite fitting Korsmeyer–Peppas, the printing of F1 and F2 modulated their release to be more controlled (zero-order), with R^2^ values, 0.998 and 0.88, respectively. Other studies show that printed tablets also had near zero-order release than the PM tablets, where EC was used in a high percentage (80%) [6]. Thus, it could be concluded that the release profile of formulations with a high level of EC (hydrophobic polymer) tends to slow down after FDM printing due to higher interactions with a hydrophobic polymer. However, the case was not the same with the more hydrophilic matrices. So FDM can either speed up drug release or slow it down based on the formulation components and SA/V as well as the thermal effect on hardness, porosity, crystallinity, and specific volume of the polymer.

## 4. Conclusions

This study focused on the effect of high temperatures applied in FDM on dissolution profile. Sustained release matrix tablets were successfully manufactured by three methods namely physical mixture, hot-melt extrusion, and 3D printing FDM. Factors such as hardness, porosity, SA/V, and crystallinity were not enough to understand the increased release for printed and HME tablets. Heating and fast cooling rate were found to reduce the true density of the polymer. The nature of the printing process seemed to create gaps within the printed tablets, which made their apparent density lower compared to HME and PM tablets. It seemed that FDM was associated with many changes that can contribute to the higher release profile of 3D printed tablets compared to conventional methods. This study showed that FDM technology can either speed up or retard release profiles based on the balance of the total charges in the characteristics of printed tablets, which are hardness, porosity, solid-state of API, and specific volume. A wider understanding of how FDM technology thermally affects polymer characteristics can help in the accurate selection of polymers to be used in extended release printed formulations.

## Figures and Tables

**Table 1:** Composition of proposed formulations and their compatibility with the FDM printer.

Formulation	Theophylline % *w/w*	EC % *w/w*	HPC JF % *w/w*	HPC EF % *w/w*	Compatibility with Gear Force in FDM Printer
F1	30	35 *	35	-	Compatible
F2	30	26.25	43.75	-	Compatible
F3	30	17.5	52.5	-	Compatible
F4	30	-	70	-	Too soft
F5	30	35 *	-	35	Too brittle
F6	30	26.25	-	43.75	Compatible
F7	30	17.5	-	52.5	Compatible
F8	30	-	-	70	Compatible

* plasticised with DBS 5% *w/w*.

**Table 2:** Crystallinity % values in all formulations prepared by the three methods.

Crystallinity %					
Theophylline		27.31%			
F1	PM	10.66	F6	PM	11.55
	HME	10.93		HME	8.46
	printed	9.27		printed	11.05
F2	PM	9.7	F7	PM	10.45
	HME	11.87		HME	9.19
	printed	8.29		printed	8.3
F3	PM	11.1	F8	PM	7.62
	HME	9.63		HME	9.24
	printed	9.6		printed	9.44

**Table 3:** Weight values of tablets made via different techniques.

Formulation	Weight (g) ± SD ^a^		
	Printed	HME	PM
F1	0.333 ± 0.017	0.333 ± 0.005	0.334 ± 0.003
F2	0.331 ± 0.012	0.332 ± 0.001	0.333 ± 0.000
F3	0.333 ± 0.011	0.335 ± 0.002	0.331 ± 0.003
F6	0.334 ± 0.007	0.333 ± 0.004	0.333 ± 0.002
F7	0.333 ± 0.01	0.333 ± 0.002	0.328 ± 0.004
F8	0.341 ± 0.021	0.337 ± 0.033	0.331 ± 0.002

^a^ SD, standard deviation from mean.

**Table 4:** SA/V values of tablets made via different techniques.

Formulation	SA/V (mm^−1^) ± SD ^a^		
	Printed	HME	PM
F1	0.81 ± 0.01	0.82 ± 0.01	0.828 ± 0.00
F2	0.81 ± 0.01	0.82 ± 0.00	0.83 ± 0.01
F3	0.82 ± 0.01	0.82 ± 0.01	0.82 ± 0.01
F6	0.82 ± 0.02	0.82 ± 0.01	0.82 ± 0.01
F7	0.82 ± 0.02	0.83 ± 0.01	0.82 ± 0.00
F8	0.82 ± 0.01	0.83 ± 0.04	0.83 ± 0.01

^a^ SD, standard deviation from mean.

**Table 5:** Hardness of tablets made via different techniques.

Formulation	Hardness (N) ± SD ^a^		
	Printed	HME	PM
F1	>550	469.84 ± 30.25	234.54 ± 12.76
F2	>550	544.98 ± 8.62	267.73 ± 16.53
F3	>550	485.65 ± 19.50	309.42 ± 3.63
F6	>550	533.21 ± 11.77	305.17 ± 13.80
F7	>550	537.97 ± 20.84	326.05 ± 7.58
F8	>550	>550	312.57 ± 21.03

^a^ SD, standard deviation from mean.

**Table 6:** The true density of tablets made via different techniques (n = 10).

Formulation	True Density (g/mL) ± SD ^a^		
	Printed	HME	PM
F1	1.222 ± 0.004	1.249 ± 0.001	1.257 ± 0.001
F2	1.206 ± 0.003	1.263 ± 0.001	1.267 ± 0.001
F3	1.213 ± 0.002	1.267 ± 0.001	1.265 ± 0.001
F6	1.219 ± 0.002	1.262 ± 0.001	1.271 ± 0.001
F7	1.227 ± 0.001	1.266 ± 0.001	1.268 ± 0.001
F8	1.217 ± 0.001	1.273 ± 0.001	1.273 ± 0.001

^a^ SD, standard deviation from mean.

**Table 7:** The apparent density of tablets made via different techniques.

Formulation	Apparent Density (g/mL) ± SD ^a^		
	Printed	HME	PM
F1	0.97 ± 0.01	1.15 ± 0.01	1.14 ± 0.01
F2	0.97 ± 0.02	1.15 ± 0.01	1.21 ± 0.04
F3	1 ± 0.04	1.16 ± 0.01	1.16 ± 0.01
F6	0.98 ± 0.04	1.16 ± 0.02	1.14 ± 0.01
F7	0.99 ± 0.05	1.18 ± 0.02	1.12 ± 0.01
F8	1.02 ± 0.05	1.18 ± 0.01	1.13 ± 0.02

^a^ SD, standard deviation from mean.

**Table 8:** Porosity of tablets made via different techniques.

Formulation	Porosity % ± SD ^a^		
	Printed	HME	PM
F1	20.33 ± 1.01	8.17 ± 1.01	9.31 ± 0.49
F2	19.39 ± 1.76	9.18 ± 0.4	6.31 ± 1.29
F3	17.56 ± 3.9	8.71 ± 0.51	8.64 ± 0.71
F6	19.26 ± 3.49	8.12 ± 1.39	10.67 ± 0.71
F7	19.05 ± 4.15	8.84 ± 0.41	11.66 ± 1.15
F8	18.19 ± 2.9	7.49 ± 1.16	10.48 ± 1.39

^a^ SD, standard deviation from mean.

**Table 9:** Dissolution efficiency of tablets obtained via different techniques.

Formulation	Dissolution Efficiency (DE) % ± SD ^a^		
	Printed	HME	PM
F1	26.98 ± 0.67	44.78 ± 0.60	35.39 ± 2.11
F2	44.30 ± 4.31	42.54 ± 5.93	32.62 ± 0.22
F3	48.99 ± 3.05	42.70 ± 2.42	40.15 ± 0.16
F6	53.88 ± 0.68	46.95 ± 0.83	33.58 ± 0.40
F7	58.49 ± 1.24	47.36 ± 0.29	42.05 ± 0.77
F8	86.87 ± 8.25	87.98 ± 3.25	80.25 ± 3.94

^a^ SD, standard deviation from mean.

**Table 10:** Water uptake of tablets made by various techniques.

Formulation	Water Uptake % ± SD ^a^		
	Printed	HME	PM
F1	60.77 ± 5.86	187.02 ± 6.02	77.5 ± 3.42
F2	153.7 ± 8.76	250.07 ± 22.92	82.4 ± 7.69
F3	197.08 ± 15.5	249.45 ± 2.35	89.32 ± 4.79
F6	294.39 ± 33.36	287.74 ± 40.15	145.36 ± 8.21
F7	378.81 ± 22.72	351.24 ± 20.56	150.32 ± 7.12
F8 *	-	-	-

^a^ SD, standard deviation from mean, * F8 was completely dissolved.

**Table 11:** Parameters of release kinetics for several models.

Formulation		PM Tablets				HME Tablets					Printed Tablets		
	Parameter	Peppas	Zero-Order	First-Order	Higuchi	Peppas	Zero-Order	First-Order	Higuchi	Peppas	Zero-Order	First-Order	Higuchi
F1	R^2^ *n*	0.997 0.53	0.720 -	0.872 -	0.996 -	0.982 0.60	0.842 -	0.967 -	0.982 -	0.998 0.66	0.998 -	0.962 -	0.962 -
F2	R^2^ *n*	0.998 0.57	0.794 -	0.910 -	0.990 -	0.998 0.64	0.876 -	0.980 -	0.973 -	0.993 0.66	0.880 -	0.985 -	0.967 -
F3	R^2^ *n*	0.998 0.60	0.839 -	0.954 -	0.982 -	0.998 0.66	0.904 -	0.979 -	0.962 -	0.998 0.64	0.848 -	0.982 -	0.979 -
F6	R^2^ *n*	0.998 0.58	0.819 -	0.925 -	0.986 -	0.997 0.59	0.831 -	0.966 -	0.984 -	0.990 0.65	0.826 -	0.988 -	0.977 -
F7	R^2^ *n*	0.998 0.60	0.812 -	0.947 -	0.987 -	0.998 0.62	0.887 -	0.980 -	0.979 -	0.994 0.67	0.885 -	0.991 -	0.969 -
F8	R^2^ *n*	0.999 1.01	0.921 -	0.984 -	0.910 -	0.987 0.96	0.900 -	0.964 -	0.914 -	0.990 0.90	0.922 -	0.971 -	0.918 -

## Data Availability

The data presented in this study are available on request from the corresponding author.
